# Herpes Zoster: Risk Factors for Occurrence, Complications, and Recurrence with a Focus on Immunocompromised Patients

**DOI:** 10.3390/diseases13030071

**Published:** 2025-02-26

**Authors:** Michał Oleszko, Paweł Zapolnik, Wojciech Kmiecik, Hanna Czajka

**Affiliations:** 1 College of Medical Sciences, University of Rzeszów, 35-315 Rzeszów, Poland; pawel.zapolnik@onet.pl (P.Z.); hanna.czajka@onet.pl (H.C.); 2 St. Louis Provincial Specialist Children’s Hospital, 31-503 Kraków, Poland; iplkmiecik@gmail.com

**Keywords:** herpes zoster, chickenpox, communicable disease, infections, immunology

## Abstract

Background: The varicella-zoster virus is a highly contagious human herpesvirus that primarily causes varicella (chickenpox) as an initial infection, targeting T cells, neurons, as well as skin cells, and can later reactivate to cause herpes zoster (shingles). Following reactivation, the varicella-zoster virus infection can lead to severe complications, the most common of which is postherpetic neuralgia. Risk factors include aging, immunosuppression, chronic diseases such as diabetes, cardiovascular disorders, respiratory conditions, and others. Objective: In this article, we present an analysis of factors increasing the risk of herpes zoster occurrence, complications, and recurrences (in particular in immunocompromised patients).

## 1. Introduction

The varicella-zoster virus (VZV), also referred to as human herpesvirus 3, is a double-stranded DNA alpha-herpesvirus responsible for causing varicella (chickenpox) and herpes zoster (shingles) [1,2].

Varicella is the clinical manifestation of a primary infection. Most people acquire VZV infection during childhood [3], usually by exposure to respiratory droplets or smears with the virus. Varicella viruses are transmissible to the respiratory tract and cause one of the most contagious diseases in humans. The virus exhibits a strong preference for human cells, targeting specific types such as epithelial cells, T lymphocytes, and neurons within ganglia [4].

VZV infection, associated with the production of infectious viral particles, occurs in permissive CD4 and CD8 T cells [5,6].

Initial viral replication takes place in the respiratory tract, after which the virus invades the regional lymph nodes. Subsequently, viremia develops, which causes a vesicular rash on the skin [7]. T cells facilitate the dissemination of the virus from the respiratory tract to the skin and neurons [8,9], as the VZV shows a preference for targeting memory T cells with some markers, like activated CD69+ T cells with the CD45RA-memory phenotype and skin-homing proteins [8].

The VZV incubation period lasts circa 2 weeks [9]. At first, prodromal syndromes may occur, especially in older children and adults, including fever, malaise, loss of appetite, headache, and mild abdominal pain [10]. A rash usually develops 1–2 days later.

After the initial infection, the VZV enters a latent state in the neural tissue. It has been identified in dorsal root ganglia, particularly in the Th3-L3 region [11], cranial nerve ganglia, and the intrinsic nervous system [12,13]. The virus can be reactivated and cause episodes of HZ, typically decades after primary infection [13].

After the virus reactivates, it replicates within neurons. Its ability to evade early immune detection during reactivation is critical for its survival and spread. The virus downregulates the key immune pathways, such as IFN signaling and MHC class II antigen presentation, which contributes to the persistence of infection in the neuronal tissues [9,14].

In the subsequent phase, the viral particles are transported from the cell bodies along the nerve axons to the associated dermatome, where the virus provokes inflammation and vesiculation [7] causing painful, erythematous, vesicular rash [3,7,15], which may affect any dermatomes, although most commonly one or two facial or thoracic dermatomes [16].

In certain older patients, pain persists even after the rash resolves, evolving into postherpetic neuralgia (PHN), which is the most frequent complication. PHN can result in significant physical impairment, emotional strain, disruption to routine activities and sleep, and may persist for more than 90 days [17,18].

The reason why PHN affects certain individuals is under scrutiny. Several factors have been studied, such as the severity of inflammation during injury, neurotoxicity, the presence of the viral strains that damage the sodium channel in nerve cells, and genetic triggers, but evidence is insufficient [18,19].

The VZV exhibits tropism to T cells, neurons, and skin cells [19]. The virus disseminates throughout the body within T cells, leading to complications, e.g., vasculitis, retinitis, and enteritis. The risk of stroke increases after a HZ episode, especially following cases with ocular involvement and in immunocompromised (IC) patients [20]. Moreover, VZV reactivation may result in cranial neuropathies, segmental motor weakness, encephalitis, or partial facial paralysis [21,22]. Moreover, the VZV can affect the temporal arteries causing giant cell arteritis, which is found to be a medical emergency, as it can lead to irreversible blindness [22,23].

Shingles oticus or Ramsay Hunt syndrome type II occurs when the VZV spreads to the vestibulocochlear nerve, potentially causing symptoms like vertigo and hearing impairment [24].

A total of 10–20% of patients with HZ suffer from herpes zoster ophthalmicus (HZO), the infection of the ophthalmic branch of the trigeminal nerve, which can cause ocular symptoms and risk of vision loss [25,26].

## 2. Occurrence and Risk Factors for HZ

HZ occurs worldwide and the season does not influence the incidence rate (IR); the characteristics of HZ are comparable in various countries all over the world [27]. The incidence of HZ varies with age ranging from 1.2 to 3.4 cases per 1000 people per year in younger patients, and increasing to 3.9–11.8 cases per 1000 individuals each year in the elderly population (i.e., over 65 years of age) [28]. These values are consistent with many studies that have estimated the burden of disease caused by HZ [9,18,29,30]. The tendency toward population aging leads to an increase in the number of people with a naturally debilitated immune system [31]. It is estimated that at least 20–30% and up to 50% of individuals who reach the age of 85 will be affected by HZ [32].

Proven risk factors for HZ include age over 50 years, immunodeficiency, acute infections, states of psychological stress [33], and female sex [27,34]. An increased risk of HZ has also been observed in individuals with diabetes [35], chronic respiratory conditions, e.g., asthma and chronic obstructive pulmonary disorder [36], cardiovascular disorders [37,38,39], and many different chronic diseases, including depression [39], rheumatoid arthritis [40,41], renal disorders [42], psoriasis [43], endocrine and metabolic disorders [44,45], inflammatory bowel disease [46], musculoskeletal disorders [44], states after a stroke [47,48] and other neurological disorders [49,50,51], digestive disorders [51], and others [37].

A Spanish-based study by Muñoz-Quiles et al. [52] provided information regarding specific conditions debilitating the immune system. The study involved 4,382,590 subjects (578,873 IC patients). The overall IR of HZ per 1000 PY was 9.15 in IC patients, 4.64 among non-IC individuals, and 5.02 in the general population. In the subgroups, the highest IR was found among recipients of hematopoietic stem cell transplant (n = 1779; IR 56.07 per 1000 PY); lower IR was observed in other conditions related to immunodeficiency including hematological neoplasia, solid organ neoplasia, HIV infection and AIDS, multiple sclerosis, rheumatoid arthritis, inflammatory bowel disease, autoimmune thyroiditis, systemic lupus erythematosus, and state after solid organ transplantations ranging from 6.13 per 1000 PY in psoriasis to 13.36 in SLE. In each of the above conditions (excluding HIV), the IR was higher in the 50–59 age group, in comparison with 18–29, 30–39, and 40–49 age groups.

The above results align with other studies assessing HZ incidences among IC patients [42,53,54]. In a German-based study by Schröder et al. (n > 1,000,000 people) [53], the IR was significantly higher (11.5 per 1000 PY, 95% CI: 11.4–11.6) in IC individuals than in non-IC patients (5,9 per 1000 PY, 95% CI: 5.8–5.9). The study provided a comparison of IR between highly IC subjects (13.4 per 1000 PY) and individuals with lower severity of immunosuppression (10.0 per 1000 PY) in both sexes and in all age groups.

As mentioned before, the HZ IR is also higher in populations with other chronic diseases. In another study by Muñoz-Quiles et al. [55] (almost 400,000 patients aged ≥50 with diabetes and a total of almost 2,300,000 individuals), the HZ IR was 9.3 cases/1000 persons per year in the diabetic population, and 6.8/1000 persons per year in the non-diabetic population, increasing with age in all groups, and was higher in all diabetes groups in comparison to non-diabetes groups.

In an American-based study by Suaya et al. [29], which enrolled 51 million people, of whom above 420,000 patients suffered from HZ, the crude incidence of HZ was 78% higher in the diabetic population compared to the non-diabetic population (7.96 cases/1000 PY vs. 4.48 cases/1000 PY); 14.5%of HZ cases occurred in patients with diabetes, while these individuals accounted only for 8.7% of the PY analyzed.

The study by Yun et al. focused on patients with autoimmune diseases [56], where systemic lupus erythematosus (SLE) emerged as the strongest risk factor for developing HZ, with the highest incidence rate of 19.9 per 1000 person-years compared to 5.3 in patients without autoimmune or diabetic conditions. Among patients aged ≥40, the age-specific IRs for rheumatoid arthritis (RA) and SLE were 1.5–2 times higher than the rates observed in healthy adults for whom HZ vaccination is recommended (8.5/1000). Additionally, elevated HZ rates have been documented in younger autoimmune patients, especially those with SLE [57].

Furthermore, the risk of HZ is influenced by the use of disease-modifying antirheumatic drugs (DMARDs). Compared to non-users, the risk was significantly higher in patients using biologic (b)DMARDs either alone or in combination with conventional synthetic (cs)DMARDs (adjusted hazard ratio [aHR] 2.53 [95% CI: 2.03, 3.16]) versus those using csDMARDs alone (aHR 1.48 [95% CI: 1.33, 1.66]; P-heterogeneity < 0.001) [58]. Among csDMARD users, the highest zoster risks were associated with cyclophosphamide (aHR 2.69 [95% CI: 1.89, 3.83]), followed by azathioprine (aHR 1.57 [95% CI: 1.07, 2.30]) and hydroxychloroquine (aHR 1.43 [95% CI: 1.11, 1.83]). In contrast, no increased risk was observed with methotrexate (aHR 1.24 [95% CI: 0.98, 1.57]), sulfasalazine (aHR 1.00 [95% CI: 0.71, 1.42]), or leflunomide (aHR 0.41 [95% CI: 0.06, 2.88]) [58].

In patients with RA, corticosteroids and Janus kinase inhibitors were associated with the highest IRs for HZ [59,60,61]. For SLE patients, the frequency of HZ was notably higher in those treated with anifrolumab compared to the placebo [62].

Moreover, individuals with other chronic diseases, like asthma, depression, coronary heart disease, chronic obstructive pulmonary disease, and rheumatoid arthritis have circa 30% higher risk of burden of HZ, in comparison to individuals without any underlying conditions [41]. In the systematic review and meta-analysis by Steinmann et al., all 21 analyzed conditions were associated with higher risks of HZ [37].

Additionally, stroke episodes are a risk factor for HZ. The cohort study by Tung et al. [48] demonstrated that stroke patients had a significantly higher risk of burden of HZ compared to the control group in the first year following a stroke episode (adjusted hazard ratio = 25.27) and over a 5-year period (adjusted hazard ratio = 3.44). Both hemorrhagic stroke and ischemic stroke caused greater risk of HZ (hemorrhagic type incidence rate ratio = 2.31, 95% CI, 1.67–3.20; ischemic type incidence rate ratio = 2.51, 95% CI 2.09–3.02). HZO was more frequent in individuals suffering from hemorrhagic stroke compared to those with ischemic stroke, while PHN was more common in patients with ischemic stroke. Risk factors for HZ occurrence are listed in Table 1.

It has been hypothesized that the burden of HZ can be triggered by COVID-19 infection by interfering with the immune system, causing a decrease in the number or dysfunction of B cells, as well as CD4+ and CD8+ T cells [63,64,65]. Moreover, COVID patients have greater psychological stress, which can also play a role in this process [66,67]. Numerous published studies have examined the connection between SARS-CoV-2 and the reactivation of latent VZV during COVID-19 [63,64,65,66,67,68,69]. However, there is no strong evidence that either COVID-19 infection or COVID-19 vaccination increases the risk of HZ burden [68,70] but, according to a United States-based observational study conducted by Bhavsar et al., individuals suffering from COVID-19, especially over the age of 50, may have up to 15% higher likelihood of developing HZ [71]. 

## 3. Recurrence of HZ

Studies comparing directly IC and non-IC patients often provide significantly important data regarding higher recurrence rates in the first subgroup [52,72,73,74,75]. However, not all comparing studies show significantly higher recurrence rates [76,77]. According to Parikh et al. [26], the estimates of the recurrence of HZ in general populations of IC or mixed (immunocompetent/immunosuppressed) populations ranged from 1.2% to 9.6% [41,74] and the IR was 1.7–16.6 of cases with HZ recurrence per 1000 PY [73,78]. For IC patients, the recurrence percentage was 0.0% [76]–18.2% [79], while the IR was 17.0 [75]–55 in [52] per 1000 PY.

In the Spanish-based study by Muñoz-Quiles [52], the overall IR of HZ recurrence per 100 PY was 2.28 in the IC cohort, compared to 1.56 in the non-IC cohort and 1.66 in the general population. In the subgroups, the highest IR was found among HSCT recipients (5.5 per 100 PY), and the lowest one in psoriasis (1.77). Moreover, these two conditions had the highest and the lowest IR of HZ, respectively.

Some other studies have analyzed the recurrence of HZ [80,81,82]. The study reporting the recurrence rate per 1000 PY was an Australian-based prospective study by Mbinta et al. (17.0 per 1000 PY in IC patients vs. 10.5 in non-IC individuals) [82].

Two time-to-event studies comparing the HZ recurrence rate between IC and non-IC patients reported significantly higher recurrence rates in IC patents [72,73].

According to the American-based study by Yawn et al. [72], the recurrence rate over an 8-year period in the IC group amounts to 12.0%. (95% CI, 5.8–17.7%), and in the non-IC group 5.7% (95% CI, 4.4–6.9%). A Korean-based study [73] by Kim et al. showed that IR in IC patients was 13.7% of total HZ incidences, while IC patients accounted for 17.8% of total HZ recurrences (in those the most were autoimmune diseases, 8.2% of the total cases), and non-IC 82.2%. The analysis by Batram et al. [41] revealed that individuals with one or more underlying conditions aged 18–49 years and 50–59 years had a higher probability of HZ recurrence compared to people without any chronic disease. Moreover, the second recurrence of HZ in these patients was 2–3x higher.

According to the study by Tseng et al. [80], the IR of recurrence amounted to 10.96 (95% CI, 10.18–11.79) per 1000 PY. The IR of recurrent HZ varied from 12.74 (95% CI, 10.90–14.90) per 1000 PY among patients aged 50–54 years to 8.92 (95% CI, 6.85–11.61) per 1000 PY among patients aged ≥80.

In a study by Qian et al. [75], during 1,846,572 PY of follow-up among 17,413 participants with an initial zoster episode (IR of 9.43 per 1000 PY; 95% PY, 9.29–9.57), 675 individuals (3.9%) suffered from a recurrence. The average time from the first and the subsequent HZ episode was 2 years for people aged 45–54 and 3 years for individuals aged 55 and older. Among patients who had a primary HZ event, the recurrence IR was 11.05 per 1000 PY (95% PY, 10.24–11.91). Recurrences were more common in women than in men, in younger individuals than older ones, and in patients with immunosuppression compared to those without.

Furthermore, multiple recurrences, although rarer, are also observed. [26]. Several works referred to this issue [26,72,74,80]. In the U.S.-based study by Yawn et al., 6.3% of participants experienced the second recurrence, and 2.1% had the third recurrence during the follow-up period [72]. In a study conducted in South Korea (746,816 analyzed patients) [73], among those with recurrences, 11.0% experienced two, and 1.2% had three recurrences. In a Canadian-based study by Russel et al., from almost 200,000 individuals with a history of HZ episodes, almost 16,000 patients (8.2%) had one recurrence and almost 4000 (2.0%) had many recurrences [81]. In a German-based study by Batram et al. [41], 25% of patients (1030 out of 4141) who had an initial recurrence experienced at least one additional recurrence over the 10-year study period. The likelihood of the second recurrence was higher than that of the first recurrence [41]. Recurrence rates and incidence rates are listed in Table 2.

## 4. Complications of HZ

The most prevalent complication of HZ is PHN. Published estimates suggest that a significant minority of patients—ranging from 5% to 30%—may also develop this condition [18,82]. PHN is characterized by severe, stabbing, and burning pain along the nerve pathway, which can last for months after the first episode of rash. Other possible complications include stroke, meningitis, myelitis, secondary bacterial infections, and scarring [3,13,18,83]. Over 30% of individuals suffering from PHN endure persistent pain for more than one year [18].

The reasons why some patients develop PHN while others do not remain unclear. Proposed factors include the severity of inflammation, the release of neurotoxic compounds, viral strains that disrupt the sodium channel activity in nerve cells, ongoing viral replication, and genetic and racial response variations [18,19]. The relative incidence of PHN that lasts more than 3 months falls within the range of 2.6% and 46.7% in the general population, 2.4% and 25.4% in the non-IC population, and 0% and 100% with comorbidities [84]. In IC patients, the relative incidence ranges from 3.9% to 33.8%, in HIV or AIDS from 6.1% to 40.2%, from 0% to 50 in depression, and from 0% to 13.8% in hematological malignances. The IR per 1000 PY is 0.15–2.1 in the general population, while in persons with comorbidities, it falls within the scope of 0.19 and 132.5 per 1000 PY (0.19–0.34 in diabetes, 132.5 in hematological malignancies, 0.42–0.48 in the IC population, 93.7 in solid organ malignances) [84]. For neuralgia lasting more than 30 days, the relative incidence is between 1.1% and 51.2% in the general population, 9.4% and 42.3% in IC individuals, 13.3% and 83.9% in comorbidities. The highest relative incidence was in persons with asthma (42.3%), depression (51.3%), HIV/AIDS (83.9%), and mixed malignancies (57.6%) [84]. The relative incidence of PHN lasting more than 180 days falls within the range of 3.6% and 32% in the general population, and 5.1–26.5% in individuals with comorbidities (e.g., 5.1 in psoriatic arthritis, 6.0–26.5% in diabetes, 6.1% in HIV or AIDS. A total of 6.6% in the IC population, 7.2% in rheumatoid arthritis, 7.0% in solid organ malignancies, and 10.2% in stem cell transplant recipients). For PHN lasting more than 1 year, the relative incidence ranges between 2.4 and 9 [84].

Ocular complications are commonly associated with HZO [84]. The IR of HZO in the general population falls within the range of 0.31 and 0.35 per 1000 PY, and the relative incidence varies between 1.4% and 15.9%. The IR is much higher in hematological malignancies (247.3 per 1000 PY) and in solid organ malignancies (218.5 per 1000 PY). A higher relative incidence is observed in individuals with HIV (up to 10.1%) and hematological malignancies (3.2% to 11.3%) [84]. In other comorbidities, the relative incidence ranges from 1.5% to 2.9% (2.5% in diabetes mellitus, 2.9% in end-stage chronic renal disease, 1.9% in rheumatoid arthritis, 1.7 in systemic lupus erythematosus, 1.6 in multiple sclerosis, and 1.5% in psoriatic arthritis) [84].

Disseminated HZ remains the most commonly observed severe complication. Its relative incidence falls within the range of 0.3% to 8.2% in the general population, 0% to 0.5% in non-IC individuals, 0–10.7 in HIV/AIDS, 1.2–14% in stem cell transplant recipients, 0–14.8% in solid organ transplant recipients, and 0.65–19.3% in hematological malignances [84,85]. In other comorbidities, such as inflammatory bowel diseases, multiple sclerosis, rheumatoid arthritis, chronic renal disease/end-stage renal disease, and systemic lupus erythematosus, the relative incidence ranges between 0% and 0.48%. The IR is 0–0.12 per 1000 PY in IC patients, 0.22 per 1000 PY in patients with RA, and 0.04 per 1000 PY in the general population. Among non-IC individuals, the IR of disseminated HZ ranges from 0.03 to 0.04 per 1000 PY, and the cumulative incidence per 1000 persons amounts to 0.004 [84].

Other cutaneous complications of HZ are bacterial superinfections, where the relative incidence is 0.1–38.2% in the general population and 3.8–6.3 in patients with HIV or AIDS [84].

In a Spanish-based study by Muñoz-Quiles [52], the overall IR of HZ complications per 100,000 PY was 14.36 in the IC cohort, compared to 2.64 in the non-IC cohort and 3.63 in the general population. In the subgroups, the IR of HZ complications ranged between 300.77 per 100,000 PY among HSCT recipients, and 2.85 per 100,000 PY in autoimmune thyroiditis.

While PHN is the most common neurological complication of HZ [22,84], stroke is one of the most dangerous [23]. According to Jiunn-Horng [23] et al., in the first year following HZ infection, patients had a significantly increased stroke likelihood compared to people without HZ (the aHR of stroke was 1.31 times higher (95% CI, 1.06 to 1.63, *p* < 0.05) after HZ and 4.28 times higher (95% CI, 2.01 to 9.03, *p* < 0.001) after HZO). The analysis of a stroke type probability demonstrated that the likelihood of ischemic stroke and intracerebral or subarachnoid hemorrhaging stroke was 1.31 higher (95% CI, 1.07 to 1.65; *p* = 0.009) and 2.79 higher (95% CI, 1.69 to 4.61; *p* < 0.001), respectively, in the first year of follow-up compared to patients without HZ. In a study conducted by Parameswaran et al. [47], in the first month after HZ burden, individuals had a 1.9 higher probability of a stroke (odds ratio, 1.93 [95% CI, 1.57–2.4], *p* < 0.0001). Among other neurological complications, the relative incidence of encephalitis was 0.02–2.7 in the general population, 0.1–0.4 in immunocompetent, and 0.05–0.42 in IC patients, while the IR per 1000 PY was 0.02–1.00, 0.01–0.1, and 0.01–0.1, respectively [84]. The nerve palsies relative incidence varied from 0.8 in the general population to 0.6 in non-IC patients, 1–1.1 in IC individuals, and 14 in solid organ transplantation recipients [84].

A nested case–control study by Chang Ku et al. [20] demonstrated that the odds ratio of stroke was significantly higher in individuals suffering from HIV after the episode of HZ [adjusted odds ratio: 1.85, 95% CI: 1.42–2.41)]. The biggest group who suffered from stroke in the study were people at the age of 35–45 and 45–54. The risk of stroke was significantly higher between 1 week and 3 months after the burden of HZ in HIV patients and was reduced gradually after the HZ infection and completely diminished after one year. Comorbidities, like hyperlipidemia, hypertension, chronic kidney disease, HCV infections, and chronic heart disease were found to be risk factors among HIV-patients [48]. Complications of HZ are listed in Table 3, while stroke as a complication is presented separately in Table 4. 

## 5. Hospitalizations Due to HZ Episodes

In the population-based retrospective study carried out in Poland by Rząd et al. regarding discharge records from 2012 to 2021 [85], the mean annual hospitalization rate for first-time HZ cases was estimated at 5.8 per 100,000 PY (95% CI: 4.8–6.7 per 100,000 PY). This hospitalization rate is consistent with the findings from other European, North American, and Pacific-Asian countries, where hospitalization rates range from 2 to 25 per 100,000 PY [18,34,86,87,88,89,90]. The hospitalization rate for HZ was also comparable to that in the Canary Islands, a Spanish island (6.99 per 100,000 PY), Italy (7–10 per 100,000 PY), and Sweden (6.9 per 100,000 PY). In Germany [85], the hospitalization rate was in the range of 13 to 108 per 100,000 PY, but the study involved the population over 50 years.

Up to 60% of patients hospitalized due to HZ or its complications are older than 60 years. In Poland [85], the mean and the median age of hospitalized patients was 64 and 69 years, respectively. In 2015 and in the years 2019–2020 [11], the length of hospital stay fluctuated between 6.8 and 7.4 days over the years. No significant differences in the length of stay were observed between the sexes. Regarding the types of HZ treated in Polish hospitals during the analyzed years, 56% of cases were uncomplicated HZ, meningitis and encephalitis accounted for 2% of cases, while disseminated HZ represented 8%. Overall, more than half of the hospitalized HZ cases were uncomplicated, though a relatively significant portion of cases could involve serious health consequences. In Spain, in the years 2016–2019 [90], the hospitalization rate was reported at 17.74 per 100,000 PY, with a mortality rate of 1.2 and a case fatality rate of 6.75%. These rates demonstrated significant increases with advancing age, among males, and in instances of complicated HZ. Among the total cases, 22.7% were related to immunosuppression, predominantly affecting individuals over the age of 65, and resulting in higher mortality rates in those aged over 80 years. The hospitalization rates are listed in Table 5.

A significant decline in hospitalizations was observed in Poland in the years 2020–2021 [86], as compared to previous years, due to the COVID-19 pandemic (*p* < 0.05). A similar situation occurred in Spain [63], where during the COVID-19 pandemic, the hospitalization rate due to HZ decreased, while the mortality rate showed an increase compared to previous years. However, this issue needs further study, as there is not enough evidence that COVID-19 influences hospitalization rates [63].

## 6. Conclusions

Our review summarizes the data regarding HZ occurrence, recurrence, and complications and underlines a higher risk of HZ and its complications, especially among IC individuals and the elderly. The burdens of HZ are more probable in these groups of patients. However, varicella is preventable through vaccination. There are currently two vaccines against HZ: live, attenuated zoster vaccine live (ZVL), licensed in 2006 [91], and recombinant zoster vaccine (RZV), available on the market since 2017 [92]. A review of the current knowledge of the zoster vaccines and the results of vaccination rates in the Subcarpathian Province in Poland is the subject of our research and will be presented in the following papers.

## Figures and Tables

**Table 1:** Risk factors for HZ occurrence.

Risk Measure	General Population	Non-IC	Comorbidity/State
Incidence rate (per 1000 PY)	5.02 [52]	4.64–5.9 [52,53,56]	IC: 9.15–11.5 [52,53] HSCT: 56.07 [52] Psoriasis: 6.13 [52] SLE: 13.36–19.9 [52,56] SOT: 12.65 [52] HM: 11.99 [52] SOM: 10.97 [52] HIV/AIDS: 12.94 [52] MS: 6.33 [52] RA: 8.5–11.05 [52,56] IBD: 8.29 [52] AT: 7.19 [52] SLE: 13.36 [52] Diabetes: 7.96–9.3 [29,55]
Adjusted hazard ratio	-	-	Stroke (1-year period): 25.27 [48] Stroke (5 year-period): 3.44 [48] Patients treated with (cs)DMARDs alone: 1.48 [58] Patients treated with (b)DMARDs and/without (cs)DMARDS: 2.53 [58] Patients with RA treated with cyclophosphamide: 2.69 [58] Patients treated with azatiophrine: 1.57 [58] Patients treated with hydroxychloroquine: 1.43 [58] Patients treated with metothrexate: 1.24 [58] Patients treated with sulfasalazine: 1.00 [58] Patients treated with leflunomide: 0.41 [58]
Incidence rate ratio	-	-	Hemorrhagic stroke: 2.31 [48] Ischemic stroke: 2.51 [48]

(b)DMARDs, biological disease-modifying antirheumatic drugs; (cs)DMARDs, conventional synthetic disease-modifying antirheumatic drugs; AT, autoimmune thyroiditis; HIV/AIDS, human immunodeficiency virus/acquired immunodeficiency syndrome; HM—hematological malignancies; HSCT, hematopoietic stem cell transplantation; HZ, herpes zoster; IBD, inflammatory bowel disease; IC, immunocompromised; MS, multiple sclerosis; PY, person-years; RA, rheumatoid arthritis; SLE, systemic lupus erythematosus; SOM, solid organ malignancies; SOT, solid organ transplantation.

**Table 2:** Recurrence of HZ.

Risk Measure	General Population	Non-IC	IC Population
Recurrence rate	1.2–12% [41,72,74]	5.7% [72]	0.0–18.2% [73,76,79]
Incidence rate (per 1000 person-years)	1.7–16.6 [73,75,78]	10.5 [82]	17.0–55 [52,75,82]

IC—immunocompromised.

**Table 3:** Complications of HZ.

Complication	Risk Measure	General Population	Non-IC	Comorbidity/State
Overall	Incidence Rate [per 100,000 PY]	3.63 [52]	2.64 [52]	IC: 14.36 [52]
Neuralgia lasting >30 days	Relative incidence [%]	1.1–51.2 [84]	9.4–42.3 [84]	Asthma: 42.3 [84] Depression: 51.3 [84] HIV/AIDS 83.9 [84] Mixed malignancies: 57.6 [84]
PHN lasting >90 days	Relative incidence [%]	2.6–46.7 [84]	2.4–25.4 [84]	IC: 3.9–33.8 [84] HIV/AIDS: 61–40.2 [84] Depression: 0–50 [84] HM: 0–13.8
PHN lasting >90 days	Incidence Rate [per 1000 PY]	0.15–2.1 [84]	-	Diabetes: 0.19–0.34 [84] HM: 132.5 [84] IC: 0.42–0.48 [84] SOM: 93.7 [84]
PHN lasting >180 days	Relative incidence [%]	3.6–32 [84]	-	PA: 5.1 [84] Diabetes: 6.0–26.5 [84] HIV/AIDS: 6.1 [84] IC: 6.6 [84] RA: 7.2 [84] SOM: 7 [84] Stem cell transplant: 10.2 [84]
Disseminated HZ	Relative incidence [%]	0.3—8.2 [84]	0–0.5 [84,85]	HIV/AIDS: 0–10.7 [84,85] SCT: 1.2–14 [84,85] SOT: 0–14.8 [84,85] HM: 0.65–19.3 [84,85]
Disseminated HZ	Incidence Rate [per 1000 person-years]	0.04 [84]	0.03–0.04 [84]	IC: 0–0.12 [84] RA: 0.22 [84]
Encephalitis	Relative incidence [%]	0.02–2.7 [84]	0.1–0.4 [84]	IC: 0.05–0.42 [84]
Nerve palsies	Relative incidence [%]	0.8 [84]	0.6 [84]	IC: 1–1.1 [84]; SOT: 14 [84]

HZ, herpes zoster; PHN, postherpetic neuralgia; IC, immunocompromised; HIV/AIDS, human immunodeficiency virus/acquired immunodeficiency syndrome; HM, hematological malignancies; SOM, solid organ malignancies; RA, rheumatoid arthritis; PA, psoriatic arthritis; SCT, stem cell transplantation; SOT, solid organ transplantation; PY, person-years.

**Table 4:** Stroke as a complication of HZ in the general population.

Complication	Risk Measure	General Population	Period
Stroke after HZ	Odds ratio	1.93 [47]	First month post-HZ
Stroke after HZ	Adjusted Hazard Ratio	1.31 [23]	First year post-HZ
Stroke after HZO	Adjusted Hazard Ratio	4.28 [23]	First year post-HZ
Ischemic stroke	Adjusted Hazard Ratio	1.31 [23]	First year post-HZ
Intracerebral/Subarachnoid hemorrhage	Adjusted Hazard Ratio	2.79 [23]	First year post-HZ

HZ, herpes zoster; HZO, herpes zoster ophthalmicus.

**Table 5:** Hospitalizations due to HZ.

Country	Hospitalizations per 100,000 PY
Overall [34,82,86,87,88,89,90]	2–25
Poland [85]	5.8
Spain (islands) [52]	6.99
Sweden [18,85]	6.9
Italy [18,85]	7–10
Germany [85]	13–108 (population aged > 50)

## Data Availability

Not applicable.

## Abbreviations

The following abbreviations are used in this manuscript:

aHR	Adjusted hazard ratio
bDMARDs	Biologic disease-modifying antirheumatic drugs
csDMARDs	Conventional synthetic disease-modifying antirheumatic drugs
CI	Confidence interval
DMARDs	Disease-modifying antirheumatic drugs
HSCT	Hematopoietic stem-cell transplantation
HZ	Herpes zoster
HZO	Herpes zoster ophthalmicus
IC	Immunocompromised
IR	Incidence rate
Non-IC	Non-immunocompromised
PHN	Postherpetic neuralgia
PY	Person-years
RA	Rheumatoid arthritis
RZV	Recombinant zoster vaccine
SLE	Systemic lupus erythematosus
VZV	Varicella zoster virus
ZVL	Zoster vaccine live
